# Supplementary material for: #ForYou? the impact of pro-ana TikTok content on body image dissatisfaction and internalisation of societal beauty standards
Source: PLoS One. 2024 Aug 7;19(8):e0307597. doi: 10.1371/journal.pone.0307597 (PMC11305550; doi:10.1371/journal.pone.0307597)
Supplement: Attachment — Submitted filename: Response to Reviewers_Final.docx [file pone.0307597.s001.docx]

**Response to Reviewers - PONE-D-23-33901**

| **Reviewer #1**  **Point #1:** The purpose of this study is not clear - why additional study is needed on the topic where many studies have already been done (as the review in the paper shows)? I agree that how social media influences body satisfaction and eating behavior is a very important topic, but making it explicit what this study added would certainly help readers. | We thank the Reviewer for this valuable feedback. We agree that the rationale for the study could be made more explicit.  We have revised the abstract and the introduction to include a more explicit description of the contribution this manuscript makes to the field. This description addresses the unique nature of TikTok as a social media platform (see p.4), highlights the unexplored links between TikTok usage and orthorexia (see p. 5), while the “Current research” section (see p. 11) has also been updated accordingly.  The ‘Theoretical and practical implications’ section of the Discussion (see p. 30) has also been modified to reflect this strengthened rationale.  While existing research has established the relationship between social media platforms such as Instagram and Twitter with body image dissatisfaction and disordered eating behaviours, the unique algorithm of the TikTok ‘For You Page’ warrants investigation, and our manuscript addresses this gap in the literature.  The present study also adds to existing literature by utilising an experimental design to examine the impact of implicit and explicit pro-anorexia content on body image satisfaction and internalisation of body ideals to support a causal relationship between pro-ana TikTok content and negative body and diet related implications. |
| --- | --- |
| **Point #2:** The introduction (with review) feels too long and repetitive. I believe requires a clearer structure. I also recommend adding a theoretical scheme of the subject that you are studying. Currently it is not clear why some study is cited in one section, and not above where this topic was already raised. | We agree with this feedback and have completely restructured the introduction to reduce repetition and streamline the flow of the arguments presented therein.  The rationale for examining body image and internalisation in the context of social media is founded on a series of relevant theoretical approaches, including social comparison theory, the transactional model, sociocultural theory, and the tripartite influence model. We believe these theoretical approaches are relevant to the topic under consideration in this manuscript and add appropriate depth to the theoretical structure of the manuscript. The newer more condensed version of the introduction offers a more cogent theoretical framework for the study. |
| **Point #3:** On page 4 TikTok is described as a platform where users have less autonomy over their newsfeed than on other platforms. I think you either need to revise this position or provide additional arguments. For example, Instagram and Twitter also have a page with recommended content which depends a lot on the content a user has interacted with previously. | Whilst it is true that other social media platforms have followed TikTok’s lead in creating their own recommended content features, this dimension of social media is most pronounced on TikTok. The ‘For You’ page is the homepage of TikTok where users spend the majority of their time on the application, compared to Instagram where the homepage is curated of content from predominantly followed accounts as well as some advertisements and suggested content.  Instagram’s explore page continues to emphasise established influencer culture by promoting accounts of public figures of influencers with large followings. Contrastingly, TikTok’s unique algorithm propels lesser-known creators into the spotlight of becoming “TikTok famous” regardless of follower count or celebrity status, which diversifies the range of content a user will be exposed to on their “For You” page.  Additional arguments regarding the TikTok “For You” page algorithm have been added to pp. 4-5 and p. 11 of the manuscript to strengthen the position that social media users have less autonomy over their newsfeed on TikTok compared to other social media platforms.  In particular, the following has been added to p.4 of the manuscript: *Whilst the popularity of TikTok’s “For You” page has prompted global leaders in social media to build their own recommended content features, this feature remains most pronounced on TikTok. The “For You” page is the homepage of TikTok where users spend the majority of their time, compared to other social media platforms where homepages consist of a curation of content from followed accounts. Instagram’s explore page continues to emphasise established influencer culture and promote accounts of public figures or influencers with large followings. Contrastingly, TikTok’s unique algorithm makes content discoverability an even playing field, as any user’s content has the potential to reach a vast audience regardless of follower count or celebrity status. TikTok users therefore have less control over their homepage newsfeed compared to other social media platforms where users elect who they follow.* |
| **Point #4:** Terminology is not always consistent - for example on page 16 you say that you study "disordered eating behaviour" but on page 17 you say "restrictive disordered eating behaviour" (the latter is probably a part of the former but it is not self-evident). | In the current study, disordered eating behaviour was measured by two separate but related constructs. Orthorexic behaviours and other dieting behaviours typical of anorexia and bulimia are measured separately by the ORTO and the EAT-26, respectively.  Throughout the study, ‘Restrictive’ disordered eating relates to dieting, oral control, and bulimic symptoms, whilst ‘healthy’ disordered eating constitutes orthorexic-like preoccupation with health food. This distinction is outlined towards the end of p.5 of the manuscript at the end of section entitled, “Orthorexia: The rise of ‘healthy’ eating pathologies”  and this terminology is consistently deployed throughout the study to differentiate orthorexic behaviours and anorexic/bulimic related behaviours. |
| **Point #5:** On page 8 you say that slim-thicc ideal may be more problematic than thin-ideal because in some countries who do not aspire to the thin ideal may be influenced more by the slim-thicc aesthetic. This argument is not clear to me. Yes, in *these* countries slim-thicc ideal might be more problematic, but not in other countries. Below that you say that slim-thicc ideal is less attainable because it requires surgeries - this seems more logical. | We have revised our argument on p. 7 of the introduction to highlight the problematic nature of the slim-thicc ideal, that is, due to the unattainability of this physique, most women require life threatening surgery to achieve this physique. |
| **Point #6:** On page 14 you cite a study about the influence of ethnicity on awareness and internalisation of ideas about thinness - not clear how why is it necessary, what argument it supports. | We agree that this is not germane to the current manuscript. This study has been removed from the manuscript. |
| **Point #7:** The link to the dataset provided only has a pdf with the tables and plots, not original data | The original data is now available on data repository, Figshare, via the following link: <https://doi.org/10.6084/m9.figshare.25756800.v1> |

| **Reviewer #2**  **Abstract:**  **Point #1:** I would appreciate it if the aim of the study were included | A statement of the study’s aim has been added to the abstract. |
| --- | --- |
| **Point #2:** The results of the comparison variables of the study in 4 groups based on average daily time on TikTok are missing in the abstract | The results of the comparison variables of daily TikTok use and disordered eating behaviours have been added to the abstract. |
| **Introduction:**  **Point #3:** I recommend rewriting the Introduction to make it 3-4 times shorter. Now, the length of this part is 14 pages. I would appreciate it if the authors presented the main research question earlier in the Introduction and then presented the specific arguments for it. The presentation of research should be more concentrated, generalization of the findings but not the presentation of separate research is recommended. | We thank the reviewer for this feedback. The introduction has undergone a full revision to improve the conciseness of expression while ensuring broader, generalised arguments are presented, as opposed to the higher levels of micro-level analysis of empirical research presented in the original submission. The length of the introduction is now nine pages, in contrast to the original 14-page version.  Signposting of the focus of the research is now included on the first page of the introduction, as follows: *In this research, we sought to explore the impact of pro-anorexia TikTok content on body image satisfaction and internalisation of beauty standards for young women. The forthcoming sections of this literature review will highlight the features of social media content that may be particularly pernicious for young female social media users and will explore disordered eating and orthorexia in a social media context, concluding with a theoretical analysis of the relationship between social media and body image and internalisation of beauty standards, respectively.* |
| **Point #4:** Presenting orthorexia, the authors are recommended to cite recent research on the state of the art of orthorexia research. Why is it important to assess orthorexia attitudes in the context of the present research? | It was important for us to assess attitudes towards orthorexia in this research because despite the frequent observation of orthorexic behaviour patterns in clinical contexts, such behaviours have received little empirical attention, while orthorexia is still not formally recognised as a psychiatric disorder. Orthorexia also acts as a socially acceptable way of regulating the body in a manner that is potentially harmful, but is in keeping with neoliberal self-improvement culture, where the body is positioned as a site of transformation and performance and this is evident on social media.    Morality and “health” discourses are often used to disguise orthorexic restrictions, yet orthorexia and anorexia nervosa share traits of perfectionism, high anxiety, the need to exert control, as well as the potential for significant weight loss. Consequently, orthorexic attitudes may provide a gateway into disordered eating behaviours under the false pretence of health and wellness consciousness.  Limited research has investigated orthorexic behaviours and TikTok, hence the current study aimed to bridge this gap in the literature. This aim has been added to the introduction (see p. 5) to ensure more clarity regarding the importance of assessing orthorexia in this study. |
| **Point #5:** The same comment is for the fitspiration content in social media. Why is fitspiration so specifically presented if it is not specifically analyzed in the research? | A large portion of fitspiration-related content has now been removed from the introduction section. The description of fitspiration content, explanation of why it can be problematic, and presentation of research about fitspiration content and mood, appearance comparison, and body image remains in the introduction.  Fitspiration is presented specifically as the ‘pro-ana’ TikTok video compilation demonstrated within the experimental condition of this study contains approximately one minute of fitspiration-style content. Fitspiration content was included within the experimental condition to represent the implicit messaging that circulates TikTok about body image ideals and eating behaviour, which similarly to orthorexic behaviour, is a wolf in sheep’s clothing insofar as pathological behaviours are disguised as health and wellbeing.  A statement has been added to the Procedure section of the Methods on p. 16 that states that fitspiration content was utilised in the pro-ana TikTok video shown to the experimental condition. This inclusion helps to clarify why fitspiration is discussed within the introduction. |
| **Point #6:** The clear aim of the study should be developed and presented, not only hypotheses. | We thank the reviewer for this feedback and have added the following to the manuscript:  On p. 5 of the manuscript: *“The current study aims to bridge this gap in the literature around TikTok use and orthorexic tendencies.”*  And in the “Aims and Hypotheses” section on p. 11: “*The current study aimed to investigate the impact of pro-ana TikTok content on young women’s body image satisfaction and internalisation of beauty standards, as well as exploring daily TikTok use and the development of disordered eating behaviour.”* |
| **Point #7:** The cross-sectional and experimental designs were used in the study, why do the authors mention only the experimental design presenting their research? Please clarify this issue in all parts of the manuscript. | The use of a cross-sectional design in the present study has been clarified and more clearly foregrounded throughout this updated version of the manuscript. |
| **Methods:**  **Point #8:** Please clearly present the Procedure explaining and dividing two parts – cross-sectional and experimental. I would advise starting the section with Procedure and then presenting Participants | The Participant and Procedure sections both explain the cross-sectional and experimental aspects of this study in a manner that is replicable for future researchers. Some further context around the cross-sectional design has been added to the Procedure section.  The analyses undertaken for the cross-sectional and experimental parts of this study are clearly outlined and delineated in the Overview section of the Results. A statement has been added to the Measures section to clarify the cross-sectional aspect of the study. In line with other cross-sectional research manuscripts published by PLOS ONE, we have chosen to leave the ordering of the Participant and Procedure sections as per the original version of the manuscript. |
| **Point #9:** The ethical aspects of the research are a big concern. It is obvious from the literature that the pro-ana content is damaging young women’s body image and well-being, however, the experiment with the damaging content (recorded video) was implemented. Is that ok from the ethical perspective? How was the possible damage compensated for study participants? | This research has undergone a full ethics review at the University at which the work was undertaken. Prior to commencement of the study, ethics approval (Approval number H21155) was obtained from the Charles Sturt University Human Research Ethics Committee (HREC) following review of a comprehensive project proposal and Human Research Ethics Application (HREA) submitted by the project team. The project proposal and HREA described the potential risks and burdens of the study to participants and how these risks would be mitigated and managed. Potential issues outlined by HREC review process were thoroughly addressed in our ethics application and evaluated by the HREC who approved the research.  Exposing some participants to pro-ana TikTok content does pose the risk of these participants experiencing psychological distress or discomfort. However, this content is readily available for any TikTok user to view and thus exposure to pro-anorexia TikTok content incurred via participation in this study for those in the experimental condition is not necessarily unique to the research context, as participants of this demographic are likely to be targeted by the TikTok algorithm to consume this content in their lives outside of this study. While there was risk of temporary distress or discomfort, the experience of prospective research participants was evaluated by the HREC who approved this research and was judged to be commensurate with participants’ social media consumption in the real-world outside of the experiment and ethically sound and ethically defensible in light of the benefits of the research.  Participants with a history of or current clinically diagnosed eating disorder were screened and excluded from the study to prevent causing distress or re-triggering disordered eating thoughts and behaviours. Participants were also forewarned of potentially distressing content prior to participation and encouraged not to participate if they were concerned about experiencing any distress given the nature of the study. Participants were also advised that they could withdraw without consequence at any time during the study. At the completion of participation, participants were issued a debriefing statement that provided eating disorder counselling and support options to manage the risk of distress or concerns about their own eating behaviours or attitudes that the study may have raised. |
| **Point #10:** The questions related to the use of TikTok and its content should be presented in a detailed way (Tables 1 and 2). It is unclear how the information presented in Tables 1 and 2 was gathered. What exact questions were asked and what possible answers were provided. It is necessary for research that might want to replicate the research in future studies**.** | The demographic questions utilised in this study have been added to the public data repository for this research (accessible via this link: <https://doi.org/10.6084/m9.figshare.25756800.v1>) |
| **Point #11:** Please exclude information in lines 516-520 since it is presented further. | These lines have been excluded. |
| **Point #12:** Is the Likert scale in EAT26 starting at 1 and ending at 6? Isn’t it 0 to 3? Please double-check the description of the instrument. | The Likert scale for the EAT26 allows participants to select one of six possible response options: *Always, Usually, Often, Sometimes, Rarely, Never*.  These six options are scored 0-3 with responses of “Sometimes”, “Rarely”, and “Never” scored as “0”, “Often” is scored as “1”, “Usually” is scored as “2”, and “Always” is scored as “3”.  The in-text description of this scale (see p. 12) has been amended as follows: *Responses to the 26-items are self-reported using a 6-point Likert scale ranging from Always (3) to Never (0).* |
| **Point #13:** Why do the authors use the ORTO-15 instrument if it lacks psychometric characteristics? I recommend excluding it from research and using more sound instruments in future studies. | A reliability analysis revealed unacceptable reliability in the present study. However, due to this unacceptable reliability, a factor analysis was performed which identified a factor relating to health food preoccupation. Another reliability analysis was performed on the five items that comprised this factor and it was found to have acceptable reliability.  This identified factor was used as the ORTO-15 measure and data from these five items were used in analyses and referred throughout the present study as ‘healthy’ disordered eating. Hence the ORTO-15 measurement utilised in the present study demonstrated adequate psychometric characteristics. |
| **Point #14:** The separate Statistical Analysis section is missing (lines 658-664; 686-693). | We have reorganised the manuscript in line with this feedback and have added this section to the manuscript (see p. 16, Line 518). |
| **Results:**  **Point #15:** Please divide the Results section presenting the cross-sectional part of the study first, and the experimental study second. Please exclude information presenting the sample from the Results part. The results of Table 1 should be integrated into the text. | We have adjusted the Results section so that the cross-sectional study findings are reported first, followed by the experimental findings. We have included further labelling of the relevant sub-sections of the Results in order to clearly differentiate between the cross-sectional and experimental findings.  A brief overview of the participant sample is presented in the Method section, with more detailed demographic analyses offered in the Results section of the manuscript. This aligns with the linear narrative of the manuscript such that it is appropriate that this information is provided after information on “Data screening” which is presented at the beginning of the Results section.  The key results of Table 1 are integrated into the text in lines 545 – 547. |
| **Point #16:** The name of the Table 2 is not appropriate. Also, it should be reconsidered if the results of the Table 2 might be presented in the text. | We have changed the name of Table 2 as follows: *TikTok #ForYou Content Demographics* to ensure the title captures the context of the data under consideration in this table.  Table 2 is titled ‘For You Page Content’ as it presents the results of content participants’ have engaged with on their TikTok For You Page (FYP). The key results of Table 2 are summarised in the text in lines 553 – 562, and 567 - 572. |
| **Discussion:**  **Point #17:** Please clearly divide between the cross-sectional part and the experimental part. I miss the results for the lines 808-811; and 924-929. | The cross-sectional part of this study is presented under its own sub-headings separately from the experimental sections in both the Results and Discussion sections.  The results of lines 808-811 and 924-929 are clearly presented in the Hypothesis Testing section of the Results in lines 576 - 596 and presented in Table 3. |
| **Point #18:** Please do not provide new results in the Discussion. | No new results have been provided in the Discussion. All results examined in the Discussion were presented in the Hypothesis Testing sections of the Results, for cross-sectional and experimental findings, respectively. |
| **Point #19:** The Discussion should also be rewritten and shortened focusing on the main research questions | The Discussion is divided by each research question so that each of the three key findings of the study are appropriately examined. The analysis provided in the Discussion is appropriate in scope and detail, with all key research findings addressed and no superfluous information included.  No change has been made to the Discussion other than to provide further clarification via sub-headings as to the discussion of cross-sectional vs. experimental findings. |
| **Point #20:** The conclusions seem too long and not specific. Please clearly state the conclusions from the cross-sectional part and the experiment. Please exclude specific practical applications from the Conclusions and write more general recommendation. | A more specific statement regarding the findings has been included in the Conclusion (see p. 33), as follows: *Our findings indicate that female-identifying TikTok users may experience psychological harm even when explicit pro-ana content is not sought out and even when their TikTok use is time-limited in nature.*  We have also removed the following statement from the Conclusion as we agree with the Reviewer that this specific argument is better placed in the “Theoretical and practical implications” section of the manuscript: *Health professionals involved in the care of women with eating disorders and other mental health issues should also be educated to ensure they are knowledgeable about the social media content their clients may be exposed to, equipping them with skills to engage in conversations about the potential detrimental impacts of viewing pro-ana and other harmful TikTok content [63].* |
